# Contrasting evolution of virulence and replication rate in an emerging bacterial pathogen

**DOI:** 10.1073/pnas.1901556116

**Published:** 2019-08-01

**Authors:** Luc Tardy, Mathieu Giraudeau, Geoffrey E. Hill, Kevin J. McGraw, Camille Bonneaud

**Affiliations:** ^a^Centre for Ecology and Conservation, University of Exeter, Penryn, TR10 9FE Cornwall, United Kingdom;; ^b^School of Life Sciences, Arizona State University, Tempe, AZ 85287-4501;; ^c^Department of Biological Sciences, Auburn University, Auburn, AL 36849-5414

**Keywords:** bacteria, emerging infectious disease, evolution of resistance, evolution of virulence, pathogen load

## Abstract

Host resistance through immune clearance is predicted to favor pathogens that are able to transmit faster and are hence more virulent. Increasing pathogen virulence is, in turn, typically assumed to be mediated by increasing replication rates. However, experiments designed to test how pathogen virulence and replication rates evolve in response to increasing host resistance, as well as the relationship between the two, are rare and lacking for naturally evolving host–pathogen interactions. We inoculated 55 isolates of *Mycoplasma gallisepticum*, collected over 20 y from outbreak, into house finches (*Haemorhous mexicanus*) from disease-unexposed populations, which have not evolved protective immunity to *M. gallisepticum*. We show using 3 different metrics of virulence (body mass loss, symptom severity, and putative mortality rate) that virulence has increased linearly over >150,000 bacterial generations since outbreak (1994 to 2015). By contrast, while replication rates increased from outbreak to the initial spread of resistance (1994 to 2004), no further increases have occurred subsequently (2007 to 2015). Finally, as a consequence, we found that any potential mediating effect of replication rate on virulence evolution was restricted to the period when host resistance was initially increasing in the population. Taken together, our results show that pathogen virulence and replication rates can evolve independently, particularly after the initial spread of host resistance. We hypothesize that the evolution of pathogen virulence can be driven primarily by processes such as immune manipulation after resistance spreads in host populations.

## Significance

With increasing antibiotic resistance, there is a pressing need to understand how host resistance naturally influences bacterial virulence and replication rates. We test this in an infection experiment using 55 isolates of a bacterium, which were collected over the course of the epidemic following its natural emergence in a North American songbird. We demonstrate virulence has increased linearly from outbreak to the present day, encompassing >150,000 bacterial generations. Despite this, bacterial replication rate only increased during the initial spread of host resistance but not thereafter. Thus, contrary to common assumptions, virulence and replication rates can evolve independently, particularly after the initial spread of host resistance.

Understanding the evolution of pathogen virulence in response to host resistance is central to predicting and managing pathogenesis (1–3). Current theory predicts a positive association between the evolution of host immunity and the evolution of pathogen virulence (4–9), with the common assumption that this positive association is underpinned by increasing replication rates in response to host resistance (5, 6, 8, 10–15). However, experimental tests of the impacts of host resistance on the evolution of pathogen virulence and replication rates, as well as the relationship between the two, remain exceptional (2, 16, 17).

In laboratory tests, host resistance can be manipulated through either vaccination with a recombinant antigen or whole-parasite immunization, with pathogen responses quantified after passage through resistant versus susceptible hosts. Using such approaches, the rodent malaria model *Plasmodium chabaudi* was shown to evolve increased virulence when repeatedly passaged through either vaccinated or immunized mice (16, 17). Parasite densities, however, only increased in vaccinated mice, with the faster growing parasites being the more virulent. Whether differences in the effectiveness of the immune responses elicited can explain differences between these findings is unknown. Regardless, these studies demonstrate that experimental increases in host resistance can drive virulence evolution, as predicted by theory; however, for some reason, this association only emerges from increased replication rates in vaccinated hosts (16, 17).

Field tests of the impacts of host resistance on pathogen virulence and replication rates are more challenging because there are few host–pathogen systems for which we have documented natural changes in host resistance and associated changes in pathogen virulence over time (18–21). Furthermore, nonresistant hosts are rarely available, but such hosts are essential for controlled experiments because changes in pathogen traits need to be measured experimentally in the absence of the confounding effects of immune activity and clearance (22). One of the few systems in which pathogens evolving in hosts of changing resistance were compared in nonresistant hosts was the myxomatosis outbreak in introduced European rabbits (*Oryctolagus cuniculus*) in Australia (21). In this case, experimental inoculation of nonresistant laboratory rabbits with 3 viral isolates collected at outbreak (in the 1950s) and 15 isolates collected >40 y later (i.e., after rabbits had become resistant) showed that virulence increased following the spread of genetic resistance (22). However, whether virulence has increased linearly or nonlinearly over time and whether increased virulence was driven by increased replication rates were not clarified.

One way of addressing these issues is to contrast measures of virulence and replication rates in a large number of distinct pathogen isolates encompassing the period before, during, and after the spread of host resistance. Here, we do so in an infection experiment using 55 distinct isolates of an emerging bacterial pathogen (*Mycoplasma gallisepticum*) of a wild bird, the North American house finch (*Haemorhous mexicanus*). *M. gallisepticum* emerged naturally in eastern US populations of house finches in 1994, after a single host shift from poultry (23, 24). In its novel finch host, this bacterial pathogen colonizes the mucosal surfaces of the conjunctiva and upper respiratory tract and causes a severe conjunctivitis, which can lead to blindness and death in the wild through starvation or predation (25, 26). As a result, its emergence in house finches gave rise to an epidemic that spread quickly and is thought to have killed millions (24, 27). In turn, the resulting intense selection pressure led house finches to evolve genetic resistance to *M. gallisepticum* within only 12 y (18, 28). Evidence for the evolution of resistance comes from 2 independent studies. First, following inoculation with a virulent 2007 bacterial isolate, house finches from disease-exposed populations displayed significantly lower bacterial loads than those from unexposed populations (18). These results cannot easily be explained by population differences other than the history of disease exposure, because finches from exposed and unexposed populations displayed equivalent gene expression in response to *M. gallisepticum* in 2000, before resistance spread in the exposed populations (18). Transcriptional responses to infection then diverged as exposed populations evolved the ability to resist pathogen-induced immunosuppression and mount a protective cell-mediated immune response (i.e., by 2007) (18, 28). Second, in a more recent inoculation experiment, we again found evidence for increased resistance in disease-exposed host populations (29). In this case, we demonstrated that host and pathogen have coevolved antagonistically since outbreak, a pattern that can only arise when hosts have evolved resistance in response to infection (30–32).

Here, we inoculated 55 distinct *M. gallisepticum* isolates collected from epidemic outbreak, throughout the initial spread of host resistance, and afterward to the present day (1994 to 2015) into nonresistant houses finches from disease-unexposed populations. While using resistant hosts would be necessary for determining the consequence of pathogen evolution on achieved pathogen load and virulence in coevolved hosts, using nonresistant hosts here is critical because tests of genetically determined changes in pathogen traits need to be conducted in a host environment in which measures are not confounded by protective immunity (16, 17, 22). Further, the isolates used were collected at random from naturally infected finches displaying natural variation in symptom severity. Given that symptoms are required for transmission, our isolates therefore comprise a representative sample of the transmitting isolates circulating during the epidemic (29). Finally, maximizing the number of pathogen isolates used, rather than using a few isolates with multiple replicates, allowed us to adopt a regression-based experimental approach specifically designed to elucidate the shape of the relationships between pathogen traits and time against background variation in hosts (33, 34). Thus, our approach allows a novel test of 1) how pathogen virulence has changed over the course of a naturally evolving epidemic; 2) how replication rates have done so; and 3) whether replication rate is positively and linearly associated with virulence, supporting the hypothesis that replication rate drives virulence evolution (5, 6, 8, 10–15).

## Results

### Effect of Year of Pathogen Sampling on Virulence.

We quantified virulence as the severity of damage done to the host, measured as the amount of body mass lost and the level of conjunctival swelling reached during the infection, as well as putative mortality rates inferred from severity of symptoms (*Methods*). All 3 measures of virulence were highly variable. For example, mass loss averaged 0.86 g (SD = 0.96 g, equivalent to a 5% reduction for an average body mass of 18.8 g) during the course of the experiment, whereas conjunctival swelling varied by 87% among individuals and 36% of birds recorded symptoms of a severity tantamount to death in the wild (25, 26, 35). The key questions are whether this variation can be explained by year of pathogen sampling and, if so, what the shape of the relationship is between year of pathogen sampling and metrics of virulence.

**Fig. 1.**
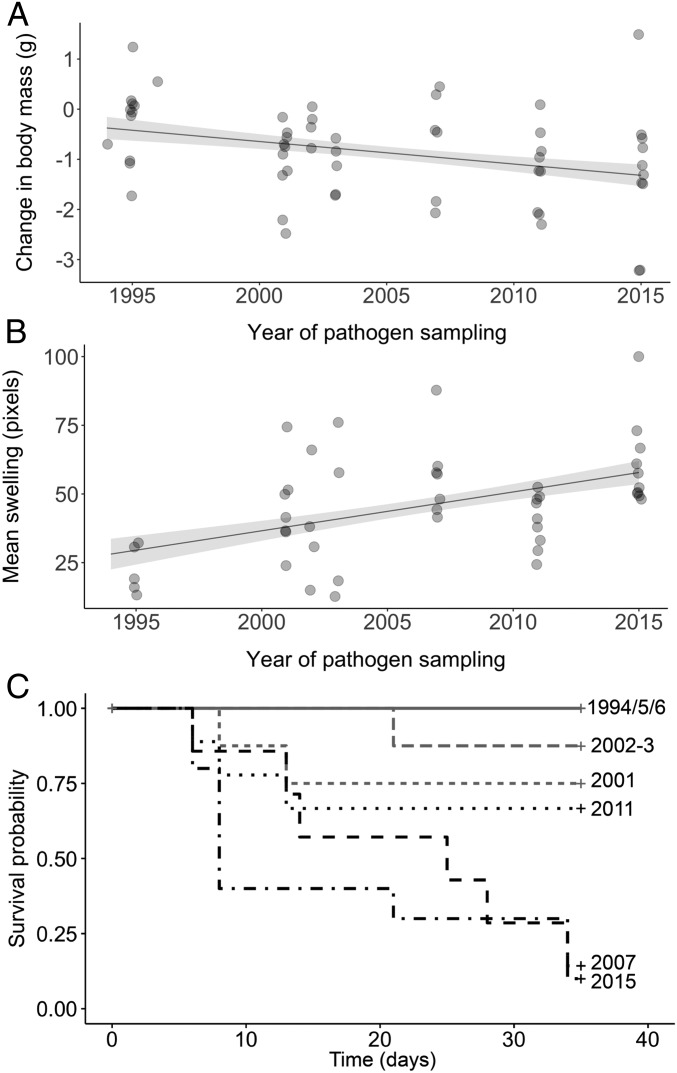
Evolution of virulence. We show body mass changes (in grams; calculated as body mass at the end of the experiment − body mass at inoculation) (*A*) and mean conjunctival swelling (in pixels) in symptomatic hosts (*B*) as a function of the year of pathogen sampling. Points represent raw values; the line is predicted from the model, with the SE represented by the ribbon. Note that some points are overlapping. (*C*) Survival probability (0/1, defined by severity of symptoms; *Methods*) over the course of the experiment (in days) for the different years of pathogen sampling (displayed on the right); isolates sampled before versus after the spread of resistance are colored in gray and black, respectively.

First, we found a significant negative association between the amount of mass lost and year of pathogen sampling, with those birds exposed to isolates sampled progressively later in the epidemic losing more mass (linear mixed model; linear year effect: estimate ± SE = −0.06 ± 0.02, *t*_53_ = −3.3, *P* < 0.002; Fig. 1*A*). This pattern was found even after controlling for significant effects of initial body mass (estimate ± SE = −0.3 ± 0.1, *t*_53_ = −2.9, *P* < 0.006); further, we found no evidence to suggest that the relationship between mass loss and year of pathogen sampling was nonlinear (quadratic year effect: estimate ± SE = 0.7 ± 0.9, *t*_50_ = 0.8, *P* = 0.42). Second, we similarly found that quantitative variation in the average conjunctival swelling of individuals also increased as a linear function of the year of pathogen sampling (linear mixed model; linear year effect: estimate ± SE = 1.4 ± 0.4, *t*_43_ = 3.6, *P* < 0.001; quadratic year effect: estimate ± SE = −10.1 ± 17.7, *t*_42_ = −0.6, *P* = 0.57; Fig. 1*B*). Finally, we found a significant effect of year of pathogen sampling on host putative survival probability (log rank test: χ^2^ = 97.1, *n* = 57, *P* < 0.0001; Fig. 1*C*), with the probability of putative survival decreasing from 100% in birds inoculated with outbreak isolates (i.e., 1994 to 1996) to 25% for those inoculated with 2007 isolates (after the initial spread of host resistance), and then to 10% in those inoculated with 2015 isolates. To elucidate whether survival probability decreased linearly with year of pathogen sampling, we analyzed how survival probabilities changed before versus after resistance initially spread in the host population. In support of a linear association, we found that the change in survival probability from pathogen emergence through the initial spread of host resistance was of comparable magnitude to the change measured over the same period of time after resistance had initially spread (logistic regression; year of pathogen sampling × sampling period interaction effect: estimate ± SE = 18.2 ± 1,883.0, *z* = 0.01, *P* = 0.99). Together, these results provide rare experimental support for the hypothesis that pathogen virulence is driven, at least in part, by increasing host resistance, and moreover suggest that virulence can continue to increase linearly over the evolutionary time period encompassed in this study (i.e., 20 y is equivalent to >150,00 bacterial generations).

### Effect of Year of Pathogen Sampling on Pathogen Load and Replication Rates.

**Fig. 2.**
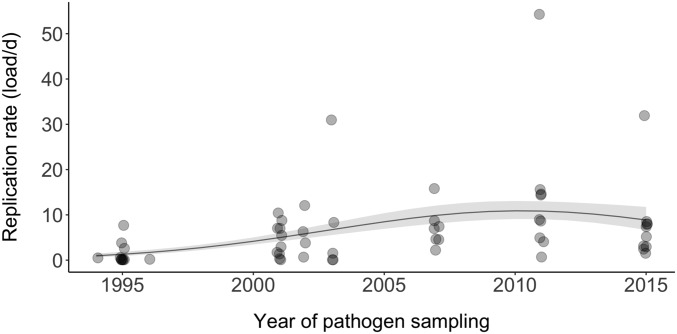
Evolution of replication rate. Replication rates (ratio of pathogen cells to host cells per day), measured as the rate at which peak pathogen load was reached at the site of infection, as a function of the year of pathogen sampling, from epidemic outbreak (1994) to over 20 y later (2015). Points represent raw values; the line is predicted from the model, with the SE represented by the ribbon. Rerunning this analysis without the 3 obvious outliers generated qualitatively comparable results and made the quadratic effect stronger (*SI Appendix*, Fig. S2*C*).

First, we investigated the function of the relationship between pathogen load and year of pathogen sampling. Pathogen load was measured as both the peak load and total load, with the latter calculated as the integral of pathogen load over the course of the 34-d experiment. The peak pathogen load averaged 83 ± 96 (SD) bacterial cells per host cell, while the total load over the experiment averaged 1,190 ± 1,440 (SD) bacterial cells per host cell. Both measures of pathogen load showed a significant positive, but quadratic, relationship with year of pathogen sampling (peak: estimate ± SE = 5.2 ± 1.1, *z* = 4.9, *P* < 0.0001 [linear effect]; estimate ± SE = −2.3 ± 1.1, *z* = −2.2, *P* = 0.03 [quadratic effect]; total: estimate ± SE = 4.9 ± 1.1, *z* = 4.4, *P* < 0.0001 [linear effect]; estimate ± SE = −2.8 ± 1.1, *z* = −2.0, *P* = 0.046 [quadratic effect]; *SI Appendix*, Fig. S1 *A* and *B*). Second, we investigated the relationship between replication rate and year of pathogen sampling. In nonresistant hosts from disease-unexposed populations, replication rate can be estimated as pathogen load divided by the time required to reach that load. However, because the timing to peak pathogen load varied among isolates (averaging 15.5 ± 8 d postinoculation [dpi]), replication rate was measured by dividing peak pathogen load by the number of days to peak load. Again, we found a significant quadratic relationship between replication rate and year of pathogen sampling (linear model; linear effect of sampling year: estimate ± SE = 5.0 ± 1.1, *z* = 4.6, *P* < 0.0001; quadratic effect of sampling year: estimate ± SE = −2.9 ± 1.1, *z* = −2.7, *P* = 0.008; Fig. 2).

### Association between Replication Rates and Virulence.

**Fig. 3.**
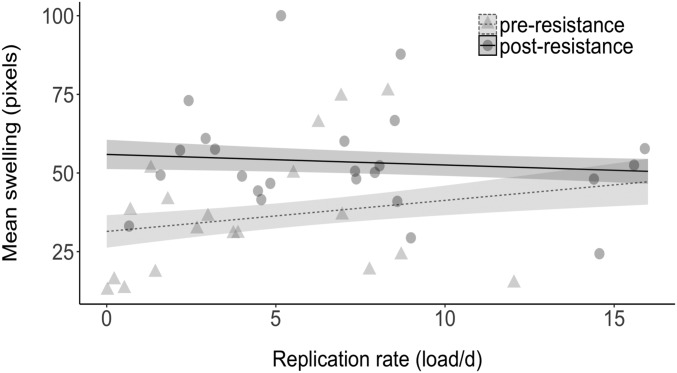
Association between replication rates (ratio of pathogen cells to host cells per day) and virulence, as measured by mean conjunctival swelling (in pixels). We show the association for pathogen isolates sampled before (in gray) versus after (in black) the spread of host resistance. Points represent raw values; lines are predicted from the model (dashed lines, isolates sampled preresistance; solid lines, isolates sampled postresistance), with SEs represented by ribbons. None of the other analyses investigating the relationship between replication rate and other measures of virulence was significant (Table 1).

A common assumption is that increasing replication rates underpin the predicted positive association between the evolution of host resistance and the evolution of pathogen virulence (5, 6, 8, 10–15). That both pathogen load and replication rate increase during the early, but not later, phase of the epidemic (Fig. 2 and *SI Appendix*, Fig. S1 *A* and *B*), while our 3 measures of virulence increased linearly throughout (Fig. 1 *A*–*C*), suggests that any relationship between replication rate and virulence will be restricted to the early phase of the epidemic at best. First, we found little evidence for a linear association between replication rates and virulence throughout the epidemic. Notably, variation in replication rates had little impact on mass loss, and there was no evidence that replication rate was associated with mean conjunctival swelling or the probability of putative survival (Table 1). Second, however, we found that this lack of association for conjunctival swelling (although not for the other 2 measures of virulence) was confounded by year of pathogen sampling. In other words, there was a positive association between replication rate and conjunctival swelling before the spread of resistance, but no significant association thereafter (analysis of covariance; replication rate × sampling period interaction effect: estimate ± SE = 1.3 ± 0.7, *t*_41_ = −2.0, *P* = 0.048; Fig. 3). These results suggest that the relationship between replication rate and virulence can be weak and that replication rate is not the primary driver of the increase in virulence following the spread of host resistance.

**Table 1.** Summary of relationships between metrics of virulence and replication rates over the course of the epidemic

Model	Estimate ± SE	Statistics	*P*	*R*^2^
1. Response: body mass change				
Replication rate	<−0.01 ± <0.01	*t*_52_ = −0.7	0.48	0.02
Replication rate^2^	0.3 ± 1.0	*t*_51_ = 0.4	0.72	0.02
Replication rate × sampling period	−0.03 ± 0.03	*t*_50_ = −1.0	0.31	0.08
2. Response: mean conjunctival swelling				
Replication rate	0.13 ± 0.31	*t*_43_ = 0.4	0.66	0.02
Replication rate^2^	−27.6 ± 19.8	*t*_42_ = −1.4	0.17	0.06
Replication rate × sampling period	1.4 ± 0.7	*t*_41_ = 2.0	**<0.05**	**0.25**
3. Response: survival probability				
Replication rate	0.07 ± 0.04	*z* = 1.6	0.10	
Replication rate^2^	−1.1 ± 2.4	*z* = −0.5	0.64	
Replication rate × sampling period	0.03 ± 0.09	*z* = 0.3	0.74	

The sampling period was categorized as before (1994 to 2004) versus after (2007 to 2015) the spread of host resistance. The single significant effect is provided in boldface.

## Discussion

Our results show that the virulence of *M. gallisepticum* has increased linearly from outbreak (1994), through the spread of house finch resistance (to 2007), to the present day (2015). Specifically, nonresistant house finches (i.e., from disease-unexposed populations) lost significantly more body mass, displayed more severe conjunctival swelling, and had lower putative survival probability when inoculated with isolates sampled at increasingly later time points in the epidemic. In contrast to linear increases in virulence, pathogen loads and replication rate displayed significant quadratic relationships with year of isolate sampling, with pathogen load and replication rate increasing from disease outbreak to the spread of host resistance, but not thereafter. Finally, we found limited evidence for an association between replication rate and our 3 measures of virulence, despite the common assumption that virulence evolution is underpinned by changing replication rates (5, 6, 8, 10–15). Together, our results support the hypothesis that increasing host resistance drives the evolution of increasing pathogen virulence, but not the assumption that this increase in virulence is mediated primarily by increasing replication rates (5, 6, 8, 10–15).

The “trade-off” hypothesis proposes that increases in pathogen fitness, represented by the number of secondary infections arising from a single infected host, can be achieved either by accelerating between-host transmission or by lengthening the duration of infection within the host, but not both (2, 5, 6, 36). One central prediction of this hypothesis is that by reducing infection duration through pathogen clearance and by alleviating the cost of virulence through reduced host mortality, host resistance should select for increased pathogen virulence (7–9). Although this prediction has been upheld in laboratory studies (16, 17), where pathogens are passaged through hosts of manipulated resistance, experimental tests of virulence responses to natural changes in host resistance are rare (20, 22). In one such test, the virulence of the myxoma virus was broadly found to increase in association with the increasing resistance of the rabbit host (37). [Although there was an initial decrease in virulence, this was likely an effect of the high virulence of the strains that were used during this eradication attempt (38).] In another test, house finch conjunctival swelling scores were found to be greater when inoculated with a 2008 isolate of *M. gallisepticum* (when resistance had spread) compared with a 1994 isolate at outbreak (20). However, our study based on 55 isolates collected before, during, and after the initial spread of host resistance provides evidence to suggest that virulence (measured in 3 different ways) has not only increased since outbreak, but has done so at a comparable rate throughout the epidemic.

Pathogen virulence is assumed to be mediated primarily through variation in pathogen replication rates (11, 13, 14, 39), so much so that pathogen load has been used as a surrogate for virulence (40). Despite this, current evidence for a tight link between replication rates and virulence is mixed (10, 14, 16, 17, 41–43). For variation in the replication rates of *M. gallisepticum* to mediate variation in virulence, both variables (replication rate and virulence) need to show equivalent patterns of response over time as hosts evolve resistance. On the contrary, however, while virulence shows a linear increase over the course of the epidemic, this is not the case for our measures of replication rate, which all showed an increase between disease outbreak and the initial spread of host resistance, but not thereafter. In other words, our measures of replication rate showed a quadratic, not linear, relationship with year of pathogen sampling. Further experiments are required to fully understand the basis of these quadratic relationships between year of pathogen sampling and replication rate or pathogen load, but there are 2 likely hypotheses for what we observed. First, replication rates may have already been optimized for pathogen transmission by the time host resistance spread in the population, with insufficient subsequent selection to drive the evolution of further increases. Alternatively, following the initial spread of host resistance, selection might have primarily operated on other fitness-maximizing traits in the pathogen, which are antagonistic to further increases in replication rates. Either way, our results suggest that replication rate is not the primary driver of the evolution of increasing virulence in *M. gallisepticum*, especially following the initial spread of resistance in house finches.

The weak associations between metrics of virulence and replication rates suggest that another pathogen trait, in addition to replication rate, accounts for significant variation in virulence. One likely candidate is the ability to manipulate the host immune system (44, 45). Evidence suggests that immune manipulation is critical to the success of *M. gallisepticum* infection (46–49). First, *M. gallisepticum* invades the mucosal surfaces of the conjunctiva and upper respiratory tract by inducing a misdirected inflammatory response, with more virulent strains inducing greater responses (46, 47, 50–55). For example, chickens experimentally inoculated with a virulent strain of *M. gallisepticum* (R_low_) display greater up-regulation of proinflammatory cytokines, which are responsible for local and systemic inflammation, and associated tissue destruction and local necrosis, than chickens inoculated with a more attenuated strain (GT5) (56). Second, the subsequent persistence of *M. gallisepticum* then depends on the bacterium’s ability to evade and suppress other immune components known to play a role in controlling *M. gallisepticum* infection (54). For example, chickens infected with *M. gallisepticum* display lower T cell activity 2 wk postinoculation than controls (52, 53), as well as lower humoral responses against other pathogens (57, 58). Similarly, we have shown previously that house finches from unexposed populations are unable to up-regulate the expression of genes associated with acquired immunity (cell-mediated immunity), again consistent with persistence being facilitated by the suppression of protective immune processes (18, 28). Finally, between-host transmission occurs through eye droplets transferred directly or left on inert surfaces as fomites, which are produced as ocular secretions resulting from inflammation (59). Thus, while the suppression of pathogen-specific immune processes is required for *M. gallisepticum* persistence, the up-regulation of nonspecific, damaging inflammatory processes is required for successful pathogen colonization and transmission. In this system, it therefore seems reasonable to hypothesize that there is independent selection on virulence and replication rate, leading to linear increases in the former, but not in the latter, over the course of the epidemic.

Our results have at least 4 important implications for host–pathogen interactions:

1) Increasing host resistance has given rise to linear increases in pathogen virulence, at least over the estimated >150,000 bacterial generations encompassed in this study.
2) By contrast, replication rates appear to have been under directional selection between pathogen outbreak and the initial spread of resistance, but not thereafter.
3) As a consequence, virulence evolution and replication rates can be under independent selection pressures, and the potentially weak associations between the 2 suggest that replication rates should not be used as a metric of virulence.
4) Finally, we hypothesize that selection on immune manipulation is dominant over that on replication rate following the initial spread of host resistance, but this hypothesis remains to be tested in this and other systems.

## Methods

### Capture and Housing.

Wild house finches from populations that have never been exposed to *M. gallisepticum* (i.e., that have not evolved genetic resistance) were captured in variety of urban and suburban sites in Arizona in the summer of 2015 (*n* = 57, 30 males and 27 females). *M. gallisepticum* has never been recorded in the sampling area despite continuous monitoring (60). Using birds that have not had the opportunity to evolve protective immune responses to *M. gallisepticum* is essential for measuring genetically determined virulence and replication rate in the pathogen without the confounds of the capacity for immune clearance (22). There is currently no evidence in this or any other system to suggest that genetically determined levels of virulence and replication rate are modified by differences in host resistance, but our ability to measure each will obviously be curtailed if done so in resistant hosts. Birds that had hatched in the spring of 2015 were trapped, weighed, and banded with a numbered metal tag for individual identification. They were then immediately transported by car to indoor aviaries at Arizona State University’s Tempe campus, where they were housed for the remainder of the experiment. On arrival, we obtained a blood sample from all birds using brachial venipuncture (60 μL of whole blood) and a choanal swab. A lack of prior infection with *M. gallisepticum* since hatching was confirmed by screening blood plasma for anti-*M. gallisepticum* antibodies using a serum plate agglutination assay (61), and a lack of current infection was verified using the choanal swabs in PCR amplification of *M. gallisepticum* DNA (62).

### Experimental Inoculation.

Each of the 55 *M. gallisepticum* isolates sampled over the course of the epidemic was inoculated into 1 bird selected at random from the 57, although 2 isolates (1 each from 1995 and 2007) were inoculated in 2 birds. Maximizing the number of pathogen isolates used is essential for clarifying the shape of the relationship between pathogen traits and time in a regression-based statistical approach (33, 34). The alternative of using fewer isolates replicated across multiple hosts would be more appropriate to fully characterize differences among pathogen isolates, but that was not the aim of the study. Further, evidence of evolution requires systematic changes in trait values over time that are observable against random background variation in ecology. In the context of our study, this random ecological variation is represented by inevitable slight among-host variation in the response to infection (although recall that none of the birds used has evolved resistance). By randomly pairing each bird with a distinct pathogen isolate (but occasional exceptions are discussed above), any slight variation in host responses to infection will be randomly distributed over the years of pathogen sampling. Thus, while the precise value of a given point will likely include some impact of host response, the shape of the regression slopes of pathogen traits over time will reflect the patterns of pathogen evolution. Finally, isolates were obtained over a 20-y period at random from naturally infected, wild-caught house finches from various urban and suburban sites in 8 different states in the eastern United States (mainly from Alabama). Given that *M. gallisepticum* requires inducing symptoms for successful transmission, our isolates are therefore a representative sample of those successfully circulating within the host population at a given time.

Isolates were obtained by swabbing the conjunctiva of a symptomatic bird and placing the swab in SP4 growth medium. Isolates were administered via 20 μL of culture containing 1 × 10^4^ to 1 × 10^6^ color-changing units per milliliter of *M. gallisepticum* in both eyes. Later quantification of the number of bacterial cells in each inoculum was determined using qPCR (discussed below), and concentrations of the inoculums were found to range from 4.1 × 10^5^ to 3.0 × 10^6^ bacterial cells per microliter (average ± SE = 1.4 × 10^6^ ± 0.6 × 10^6^ bacterial cells per microliter). To account for any variation in the number of bacterial cells inoculated (i.e., dose), we verified that there was no correlation between dose and year of sampling of the isolate (Spearman’s rank correlation: *P* = 0.49), and we included dose as a covariate in all our analyses (*Statistical Analyses*). None of the isolates had been passaged in culture more than 3 times (63). All 57 birds were maintained individually in separate cages with ad libitum food and water from the time they were inoculated and throughout the duration of the 34-d experiment. The experiment was stopped at 35 dpi, and all birds were euthanized as stipulated by home office licensing. Protocols were approved by Institutional Animal Care and Use Committees of Arizona State University (permit 15-1438R), as well as by Institutional Biological Use Authorizations to Auburn University (BUA 500), and by the University of Exeter’s Ethics Committee.

### Symptom Severity.

We have shown previously that mass loss is indicative of the severity of infection in nonresistant birds from unexposed populations, and so can be used as a measure of virulence (64). All birds were weighed (±0.01 g) at the start and end of the experiment using a top-pan balance. To quantify the size of the conjunctiva, and so the severity of conjunctival swelling, we photographed the right and left eyes at 0, 6, 13, and 25 dpi from a standardized distance. We then measured the average area of the conjunctiva swelling across the 2 eyes and at each day as follows: the area of the outer ring minus the area of the inner ring at 6, 13, or 25 dpi − the area of the outer ring minus the area of the inner ring at 0 dpi (65). Measurements of photographs were done blindly with respect to the isolate inoculated. Finally, eyes were also inspected visually on days 3, 6, 8, 14, 21, 25, 28, and 34 postinfection: Infection is considered lethal when the conjunctiva is red to purple and the eye is difficult to see and produces discharge. Such symptoms, with little or no vision possible, are thought to have caused the death of millions of infected finches due to starvation or predation (25, 35, 62).

### Bacterial Load.

Bacterial load was measured from conjunctival and tracheal swabs obtained at 8, 14, 21, and 28 dpi by quantifying the number of *M. gallisepticum mgc2* gene copies and the number of house finch *rag1* gene copies using a redesigned qPCR assay (assay design, validation, and details are shown in *SI Appendix*, Tables S1 and S2). Pathogen load was then determined as the number of *M. gallisepticum* cells divided by the number of house finch cells to control for variation in sampling efficiency (66). DNA was extracted using a QIAGEN DNeasy Blood and Tissue Kit according to the manufacturer’s standard protocols.

### Statistical Analyses.

All statistical analyses were conducted in R version 3.3.2 (67), and figures were made using ggplot2 (68). We verified that any variation in the number of bacterial cells inoculated (i.e., dose) did not confound our results. There was no correlation between the dose inoculated and the year of pathogen sampling (as discussed above), and dose was not a significant covariate in any of our analyses (all *P* > 0.5). These results show that any slight variation in dose inoculated was not systematically biased toward isolates of high virulence, and that it was not sufficient to confound any of our results.

#### Virulence.

Analyses of mass loss and conjunctival swelling were conducted using linear models with normal error structures, with dose inoculated and year of pathogen sampling fitted as fixed terms. For changes in body mass, we also included initial body mass at inoculation as a covariate. Putative survival probability over time was analyzed using a log-rank test with year of pathogen sampling and dose inoculated as explanatory terms. Further, differences in temporal changes in survival probability from outbreak to the spread of host resistance versus an equivalent period of time after resistance had spread were modeled using a logistic regression with survival (0/1) as the response variable, and with year of pathogen sampling, sampling period (pre- vs. postresistance), their interaction, and dose inoculated as explanatory terms.

#### Pathogen load and replication rate.

Most isolates achieved a low pathogen load and showed relatively low rates of replication, although some displayed substantial levels of each. As a consequence, these data followed a negative binomial distribution, and so were analyzed using generalized linear models with negative binomial error structures and logarithm link functions (69, 70), and with dose inoculated, year of pathogen sampling, and year of pathogen sampling^2^ fitted as fixed terms. It is important to note that such log-link functions do not log-transform the response term, but exponentiate the explanatory term. As such, quadratic relationships between year of pathogen sampling and measures of pathogen load and replication rate are not expected by chance in these models.

#### Association between replication rate and virulence.

To test for associations between replication rates and virulence, we ran linear models with either body mass change or conjunctival swelling as the response variable and logistic regressions with survival (0/1) as the response variable. Potential explanatory terms included were replication rate, replication rate^2^, and the interaction between replication rate and sampling period (pre- vs. postresistance). Dose inoculated was fitted as a covariate, but this was never significant.

## Supplementary Material

Supplementary File
