# Supplementary material for: Contrasting evolution of virulence and replication rate in an emerging bacterial pathogen
Source: Proc Natl Acad Sci U S A. 2019 Aug 1;116(34):16927–32. doi: 10.1073/pnas.1901556116 (PMC6708350; doi:10.1073/pnas.1901556116)
Supplement: Supplementary File [file pnas.1901556116.sapp.pdf]

SI Appendix

Tardy et al.

RESULTS

Figure S1. Evolution of pathogen load and replication rates. (**A**) Total pathogen load (ratio of pathogen cells to host cells), measured as the integral over the course of the experiment; (**B**) Peak pathogen load (ratio of pathogen cells to host cells), as a function of the year of pathogen sampling, from epidemic outbreak (1994) to over 20 years later (to 2015). Points represent raw values; the line is predicted from the model with the standard error represented by the ribbon. Re-running these analyses without the 2 obvious outliers in (A) and (B) generated qualitatively comparable results, and made the quadratic effects stronger (see below Figs. S2A-B).

A

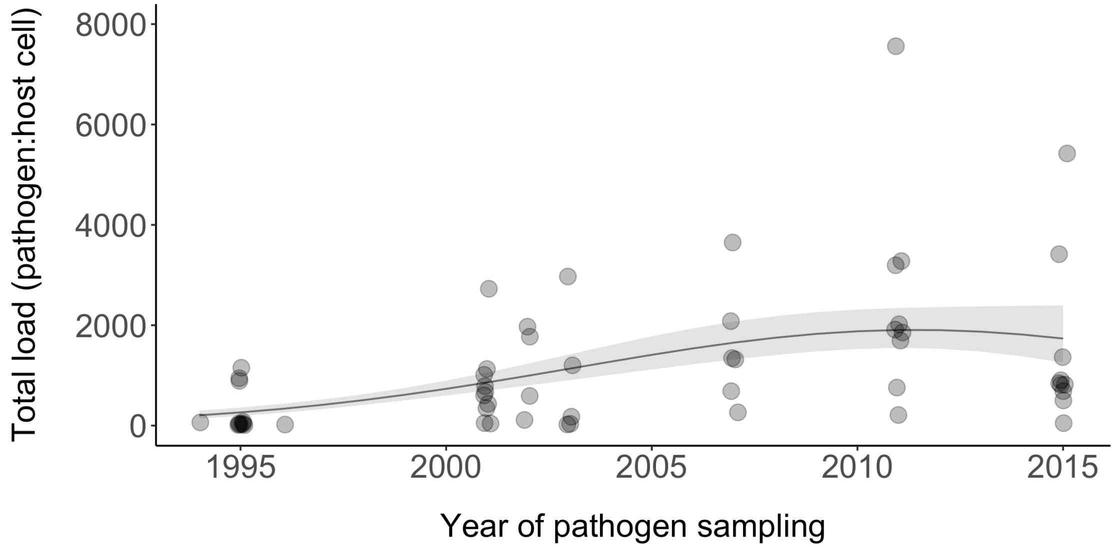

B

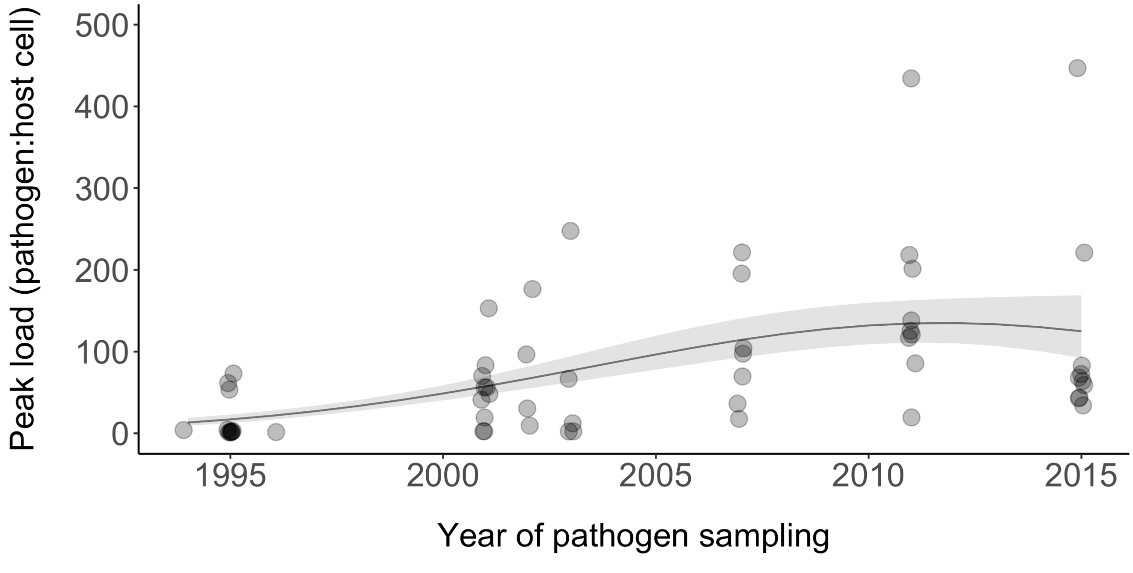

www.pnas.org/cgi/doi/10.1073/pnas.1901556116

Removing outliers

We re-ran analyses investigating the changes in pathogen load (total and peak) or replication rate over the course of the epidemic without the inclusion of 2 obvious outliers in Fig. S2A and Fig. S2B, and the 3 outliers in Fig. S2C. The results generated were qualitatively comparable, and evidence for a quadratic relationship was even stronger. Indeed, both measures of pathogen load showed a significant positive and quadratic relationship with year of pathogen sampling (*Peak*: estimate ± se = 3.9 ± 1.1, z = 3.6, p < 0.0004 (linear effect); estimate ± se = -2.7 ± 1.1, z = -2.5, p = 0.014 (quadratic effect); *Total*: estimate ± se = 4.1 ± 1.0, z = 4.0, p < 0.0001 (linear effect); estimate ± se = -2.8 ± 1.0, z = -2.7, p = 0.007 (quadratic effect); Figs. S2A, B). In addition, we found a significant quadratic relationship between replication rate and year of pathogen sampling (linear model; linear effect of sampling year: estimate ± se = 3.9 ± 1.0, z = 4.1, p < 0.0001; quadratic effect of sampling year: estimate ± se = -2.7 ± 0.9, z = -2.9, p = 0.004; Fig. S2C).

Figure S2. Evolution of pathogen load and replication rates after removing 2-3 outlying values (see above). (**A**) Total pathogen load (ratio of pathogen cells to host cells), measured as the integral over the course of the experiment; (**B**) Peak pathogen load (ratio of pathogen cells to host cells); and (**C**) Replication rates (ratio of pathogen cells to host cells/day), measured as the rate at which peak pathogen load was reached at the site of infection, as a function of the year of pathogen sampling, from epidemic outbreak (1994) to over 20 years later (to 2015). Points represent raw values; the line is predicted from the model with the standard error represented by the ribbon.

A

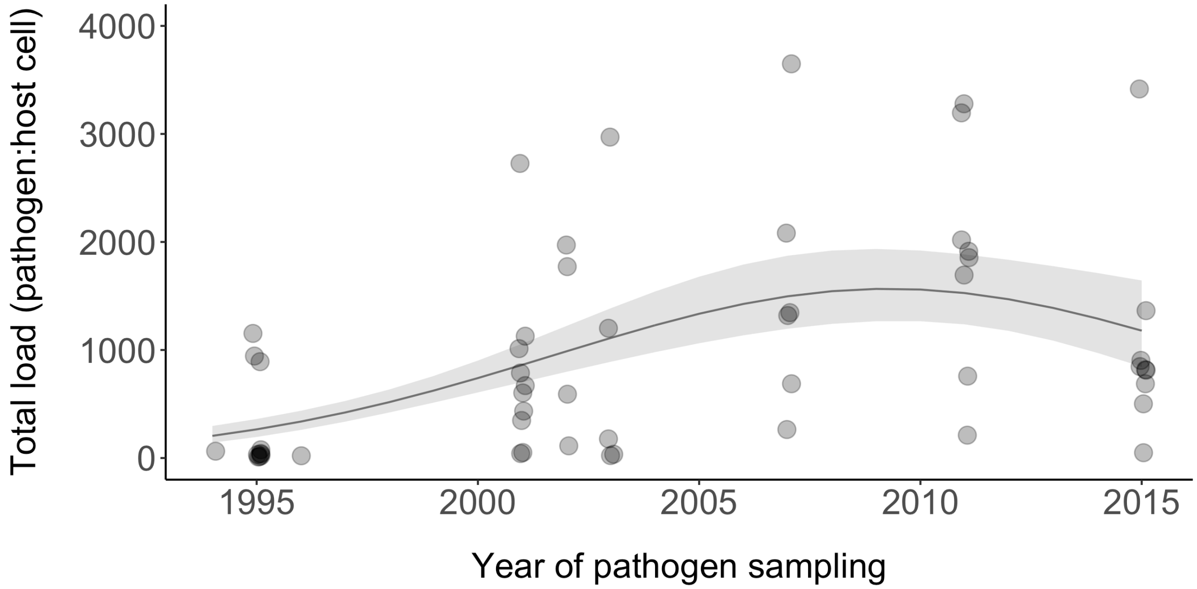

B

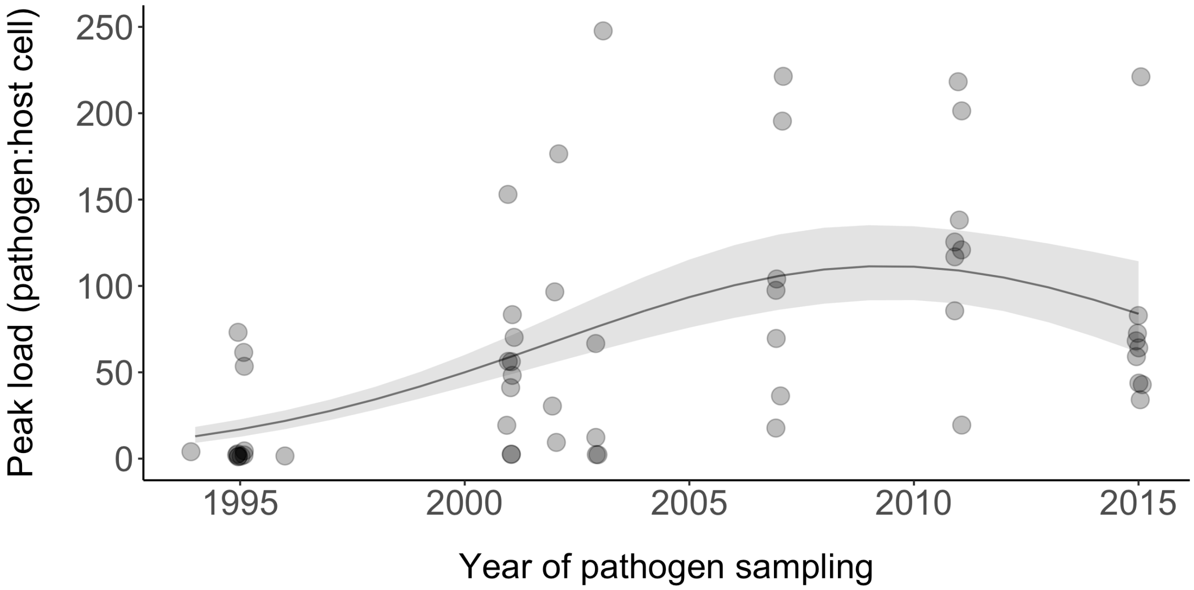

C

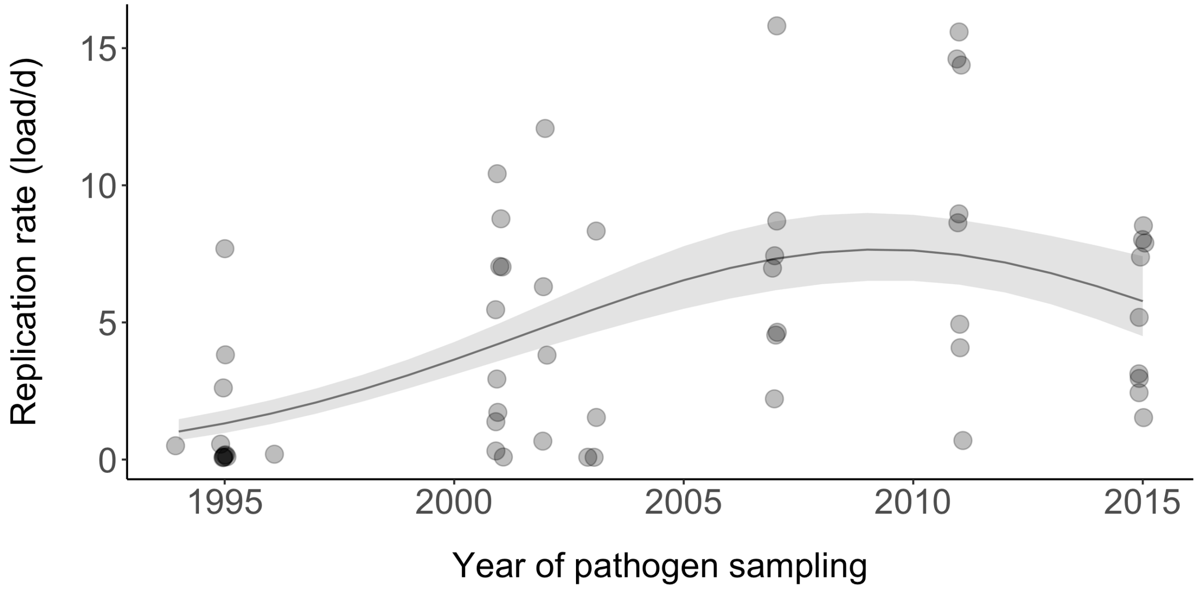

METHODS

Capture, housing and experimental inoculation

At capture, although none of the birds displayed any sign of infection with other diseases, all birds were prophylactically medicated for infection with *Trichomonas gallinae* with carnidazole (Spartrix, Janssen/Elanco) and *Isospora spp* with sulfadimethoxine over 40 days. The birds were then allowed to acclimate for one month prior to experimental onset and provided with *ad libitum* food and water throughout.

Bacterial load

The gene *mgc2* encodes a cytadhesin protein and is present at one copy/*M. gallisepticum* genome, while the recombination-activating gene *rag*1 is present in two copies/diploid house finch cell (66). We re-designed and optimised a qPCR assay for the single-copy *M. gallisepticum mgc2* and *H. mexicanus rag1* genes(1), with the aim of improving amplification specificity and efficiency. Notably, our assay includes: (1) shorter amplicons for faster amplification; (2) primers and probes designed for near identical thermodynamics between *mgc2* and *rag1* in order to promote high efficiencies in multiplex reactions; (3) lower background signal due to refined fluorophores and quenching.

We designed oligonucleotide probes and primers from consensus assemblies of *M. gallisepticum mgc2* gene sequences (Genbank IDs: CP003513.1, CP003512.1, CP003511.1, CP003508.1, CP003507.1, CP003509.1, CP003506.1, CP003510.1) and of *H. mexicanus* (and other closely related *Haemorhous*/*Carpodacus* species) *rag1* gene sequences (Genbank IDs: EU165349.1, EU165350.1, KJ455991.1, KJ455990.1, KJ455989.1, KJ455986.1, KJ455985.1, KJ455992.1, KJ455988.1), using Geneious™R8 v.8.1.8 (2) and Primer3 (3), and checked for specificity with Primer BLAST (4) (see Table S1 and S2 for sequences and details of oligos). Standard curves for both *mgc2* and *rag1* amplicons were produced by cloning of approx. 600bp gene fragments from *M. gallisepticum* strain R_{Low} and from an uninfected Arizona population control bird respectively into separate pCR™2.1 plasmid vectors in *Escherichia coli*, using an Invitrogen™ TA Cloning™ Kit according to manufacturer standard protocols. Plasmids containing target sequences were validated by restriction endonuclease analysis and checked for specific binding of internal primers by PCR and gel electrophoresis. 10-fold dilution series of plasmids containing either *mgc2* or *rag1* target sequences were quantified using a Qubit™ dsDNA HS Assay Kit, and their accuracies and efficiencies further verified by qPCR as individual and multiplexed reactions. The final range of standards used in experiments was approx. $1.6x10^8 – 1.6x10^3$ *mgc2* target copies and $8.0x10^7 – 8.0x10^2$ *rag1* target copies (Fig. S3). Limits of detection and quantification were determined by extinction dilution of standards (5), with limits of quantification set to a threshold coefficient of variation (CV) of 35%, representing absolute values of 28 target copies for *mgc2* and 18 target copies for *rag1*.

Multiplex qPCRs for *mgc2* and *rag1* were conducted using an Applied Biosystems™ StepOnePlus™ Real-Time PCR system. Multiplex qPCRs for *mgc2* and *rag1* were each run in a final volume of 20 µl and contained: 2µl of either plasmid standard or sample genomic DNA template, 1 µl each of 10 µM mgc110-F/R and rag1-102-F/R primers (total 4ul; Table 2), 0.5 µl each of 10 µM Mgc110-JOE and Rag1-102-FAM fluorescent hydrolysis probes (total 1 µl), 10 µl of 2X qPCRBIO Probe Mix HI-ROX (PCR BIOSYSTEMS) and 3 µl Nuclease-free water (Ambion®). Cycling conditions were as follows: 95°C for 3 minutes for initial denaturing of template DNA, followed by 45 cycles of 95°C for 1 second and 60°C for 20 seconds for primer and probe binding and amplification of target DNA. Sample concentrations were determined by

comparison to standard curves of both *mgc2* and *rag1* amplicons. Samples and standards were measured in duplicate in each run with a negative control of elution buffer. All data was exported to LinRegPCR v.2017.1 for calculation of individual reaction efficiencies and quantification of low-amplification samples (67, 68). Between-run variation was normalised using Factor qPCR v.2016.0 (69), with standard series presented as between-run replicates.

Table S1: Primers and probes used in the quantification of bacterial load. 6FAM/JOE- fluorophore moieties for probes. BHQ1 – Black-hole quencher 1. [+N] – modified LNA base.

**Name**	**Sequence**	**Use**
Rag1-102-F	5'-GCCCTCCTACCAGGTTATCA-3'	Forward primer *rag*1
Rag1-102-R	5'-TGGCAGTCCTGATAGTCCAT-3'	Reverse primer *rag*1
Rag1-102-FAM	5'-[6FAM]-TTGAGTGGAAACCTC[+C][+C][+C]TGA-[BHQ1]-3'	Probe *rag*1
Mgc110-F	5'-AATGCCACCAAGACCAAACT-3'	Forward primer *mgc*2
Mgc110-R	5'-CAGCTTTATTTCCCATCGGC-3'	Reverse primer *mgc*2
Mgc110-JOE	5'-[JOE]-[+A]ACCAAGACCAGGTTTC[+A]GAC-[BHQ1]-3'	Probe *mgc*2

Table S2: Primers and probes used in the quantification of bacterial load. 6FAM/JOE- fluorophore moieties for probes. BHQ1 – Black-hole quencher 1. [+N] – modified LNA base.

Name	Sequence	Target	Use
Rag1-608-F<br>Rag1-608-R	5'-TCATCCTGCTGTCTGTCTGG-3'<br>5'-GATCCGATTCATCAGCCAGC-3'	608bp fragment of *rag1* from *H.mexicanus* genomic DNA	Construction of pCR™2.1 – *rag1* plasmid for standard curves
Rag1-102-F<br>Rag1-102-R	5'-GCCCTCCTACCAGGTTATCA-3'<br>5'-TGGCAGTCCTGATAGTCCAT-3'	Internal sequence of pCR™2.1-*rag1* plasmid	Amplification of *rag1* target for probe binding in qPCR
Rag1-102-FAM	5'-[6FAM]-TTGAGTGGAAACCTC[+C][+C][+C]TGA-[BHQ1]-3'	Internal sequence of Rag1-102 amplicon	Quantification of Rag1-102 amplicon in multiplex qPCR
Mgc2-597-F<br>Mgc2-597-R	5'-GGTGCTGGGTTGATTGTTGT-3'<br>5'-GTGATTAAACCCACCTCCAGC-3'	597bp fragment of *mgc2* from genomic *M. gallisepticum* DNA	Construction of pCR™2.1 – *mgc2* plasmid for standard curves
Mgc110-F<br>Mgc110-R	5'-AATGCCACCAAGACCAAACT-3'<br>5'-CAGCTTTATTTCCCATCGGC-3'	Internal sequence of pCR™2.1-*mgc2* plasmid	Amplification of *mgc2* target for probe binding in qPCR
Mgc110-JOE	5'-[JOE]-[+A]ACCAAGACCAGGTTTC[+A]GAC-[BHQ1]-3'	Internal sequence of Mgc2-110 amplicon	Quantification of Mgc2-110 amplicon in multiplex qPCR

Further validation of plasmid standards was made by comparison of amplification efficiencies between plasmid DNA and genomic DNA template serial dilutions, as well as with genomic DNA spiked with known quantities of plasmid DNA.

(1) *Serial dilution and cycle-based efficiency calculations:* StepOne™ Software v2.3 (ABI) reported typical efficiency values of 92.7% and 91.7% respectively for *rag1* amplification from plasmid DNA and genomic DNA templates and 96.4% and 94.3% respectively for *mgc2* amplification from plasmid DNA and genomic DNA templates. Additionally, LinRegPCR calculated mean efficiencies (across multiple runs and replicates) for *mgc2* from genomic DNA at 83.3% (n=1137), for *mgc2* from plasmid DNA at 83.2% (n=128), for *rag1* genomic DNA at 85.3% (n=1137) and for *rag1* from plasmid at 85.2% (n=128). N.B. LinRegPCR typically produces lower efficiency values than other methods due to

differences in efficiency calculations and the greater variation in efficiencies observed when measuring reactions independently.

(2) Genomic DNA + plasmid spike tests: Amplification efficiencies of *mgc2* and *rag1* from either plasmid or genomic DNA was further verified by spiking dilutions of genomic DNA with known quantities of plasmid DNA targets. 10-fold dilutions of house finch or *M. gallisepticum* genomic DNA were measured independently and when spiked with known quantities of pCR2.1-rag1 or -mgc2 plasmid (827 *rag1* and 1709 *mgc2* copies respectively). Accuracy was determined by linear regression of observed copy number against expected copy number (genomic DNA + plasmid) in each reaction. *rag1* amplification from genomic DNA spiked with plasmid DNA observed *vs*. expected copy number coefficient = 1.03 ± 0.04, *mgc2* amplification from genomic DNA spiked with plasmid DNA observed *vs*. expected copy number coefficient = 1.00 ± 0.006, indicating that amplification efficiencies are analogous between plasmid or genomic DNA templates.

Figure S3: Linearity of **(A)** *mgc2* and **(B)** *rag1* standard curves from linear regression of standard dilution series (n=32) copy number against factor-corrected starting concentrations calculated using LinRegPCR.

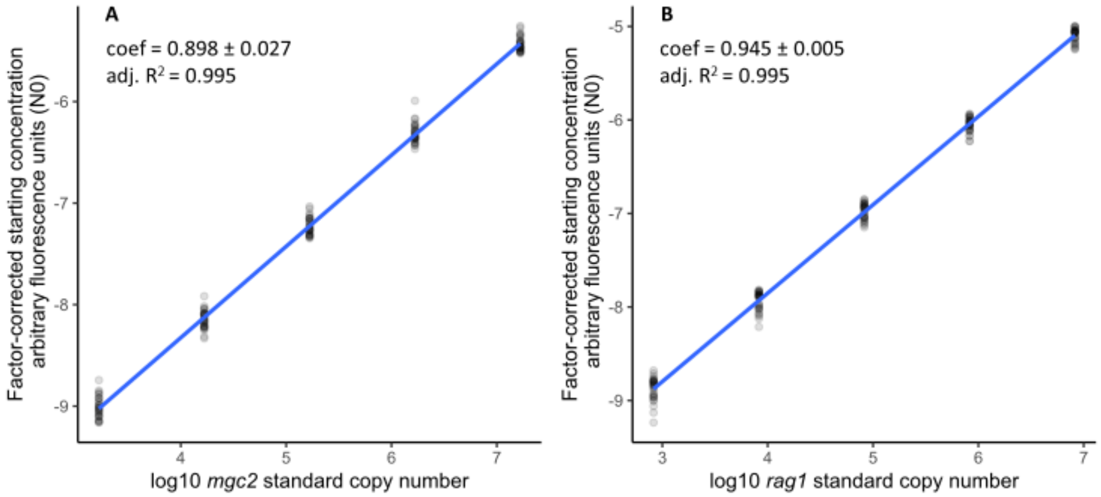

Literature

1. Grodio JL, Dhondt KV, O'Connell PH, & Schat KA (2008) Detection and quantification of Mycoplasma gallisepticum genome load in conjunctival samples of experimentally infected house finches (Carpodacus mexicanus) using real-time polymerase chain reaction. *Avian Pathology* 37(4):385-391.

2. Kearse M*, et al.* (2012) Geneious Basic: An integrated and extendable desktop software platform for the organization and analysis of sequence data. *Bioinformatics* 28(12):1647-1649.
3. Untergasser A*, et al.* (2012) Primer3-new capabilities and interfaces. *Nucleic Acids Research* 40(15):12.
4. Ye J*, et al.* (2012) Primer-BLAST: A tool to design target-specific primers for polymerase chain reaction. *Bmc Bioinformatics* 13:11.
5. Forootan A*, et al.* (2017) Methods to determine limit of detection and limit of quantification in quantitative real-time PCR (qPCR). *Biomolecular Detection and Quantification* 12:1-6.
